# Nanoscale imaging of charge carrier transport in water splitting photoanodes

**DOI:** 10.1038/s41467-018-04856-8

**Published:** 2018-07-16

**Authors:** Johanna Eichhorn, Christoph Kastl, Jason K. Cooper, Dominik Ziegler, Adam M. Schwartzberg, Ian D. Sharp, Francesca M. Toma

**Affiliations:** 1 Chemical Sciences Division, Lawrence Berkeley National Laboratory, 1 Cyclotron Road, Berkeley, CA 94720 USA; 2 The Molecular Foundry, Lawrence Berkeley National Laboratory, 1 Cyclotron Road, Berkeley, CA 94720 USA; 3 Scuba Probe Technologies LLC, 255 Lina Ave, Alameda, CA 94501 USA; 4 Walter Schottky Institut and Physik Department, Technische Universität München, Am Coulombwall 4, 85748 Garching, Germany

## Abstract

The performance of energy materials hinges on the presence of structural defects and heterogeneity over different length scales. Here we map the correlation between morphological and functional heterogeneity in bismuth vanadate, a promising metal oxide photoanode for photoelectrochemical water splitting, by photoconductive atomic force microscopy. We demonstrate that contrast in mapping electrical conductance depends on charge transport limitations, and on the contact at the sample/probe interface. Using temperature and illumination intensity-dependent current–voltage spectroscopy, we find that the transport mechanism in bismuth vanadate can be attributed to space charge-limited current in the presence of trap states. We observe no additional recombination sites at grain boundaries, which indicates high defect tolerance in bismuth vanadate. These findings support the fabrication of highly efficient bismuth vanadate nanostructures and provide insights into how local functionality affects the macroscopic performance.

## Abstract (web summary)

The performance of energy materials is affected by structural defects, as well as physicochemical heterogeneity over different length scales. Here the authors map nanoscale correlations between morphological and functional heterogeneity, quantifying the trap states limiting electronic transport in bismuth vanadate thin films.

## Introduction

Photoelectrochemical (PEC) water splitting is a promising approach to simultaneously capture and store renewable solar energy. It has the advantage of producing energy-dense fuel with minimal carbon footprint without competing against food stocks^1,2^. However, PEC systems must target high solar-to-hydrogen efficiency, low production cost, and long device lifetimes^3–6^. In this context, a promising semiconductor light absorber is the monoclinic scheelite-phase bismuth vanadate (BiVO_4_)^7–11^. BiVO_4_ absorbs visible light^12^, has favorable band alignment for water oxidation^13–15^, and possesses relatively long photocarrier lifetimes^13,16^. However, its PEC performance is limited by poor majority carrier transport, deviations from ideal stoichiometry, and defects^16–19^. As a consequence, the experimentally measured solar-to-hydrogen conversion efficiency of BiVO_4_ falls far short of its theoretical limit because of charge trapping in the bulk and at interfaces, and/or (photo)corrosion processes^9,20^.

These functionality and stability issues are common to several PEC materials and may be strongly correlated with nanoscale chemical and physical heterogeneity^21,22^. Currently, the onset potentials and saturation photocurrents of PEC materials are analyzed by cyclic voltammetry to estimate their PEC performance. However, these macroscopic measurements do not provide information about local performance variations within the sample. In thin-film semiconductors, the performance and efficiency may be significantly influenced by grains, grain boundaries, and crystal facets. In this respect, BiVO_4_ is an exemplary model material, since it is typically synthesized as a polycrystalline material with a grain structure, and it is used here as a platform to elucidate the influence of local functional variations at the nanoscale. In addition, previous studies indicated variations in the electrochemical functionality of different single crystal facet orientations^23–25^. However, understanding the interplay between morphological variations and local photocurrents through nanoscale characterization of structure-function relationships still needs to be addressed. Elucidation of such loss processes and charge-transport limitations at the nanoscale can provide a foundation for improving the functionality by engineering tailored hierarchical assemblies with controlled composition, defects, and disorder.

Photoconductive atomic force microscopy (pc-AFM) is a versatile characterization method with nanoscale resolution for correlating structural and optoelectronic properties. In general, pc-AFM can provide (photo)current maps that reveal nanoscale, (opto)electronic heterogeneity across the film, as well as single current–voltage curves (*IV*-curves) that provide insights into the charge-transport mechanism. Conductive mapping is already an established technique to analyze local charge transport and optoelectronic properties of solar cell materials such as hybrid halide perovskites^26–28^ and CdTe^29^. For this type of measurement, the solar cell top contact is replaced by a metal-coated AFM probe. However, to the best of our knowledge, the use of pc-AFM to investigate the functional heterogeneity and to understand the charge carrier transport limitations at the nanoscale in photoelectrochemical materials such as metal-oxide semiconductors still needs to be fully explored. For pc-AFM, the solid/liquid interface is substituted by a solid/solid interface, namely the semiconductor/probe contact.

Here, we use pc-AFM to study nanoscale optoelectronic heterogeneity in BiVO_4_ thin-film photoanodes and provide insights into the charge-transport limitations in this material. We observe a non-uniform current distribution across BiVO_4_ thin films with higher photocurrent at facet planes and facet boundaries with respect to grain boundaries. The associated image contrast strongly depends on the semiconductor/probe contact. In addition, dark and photocurrent maps show no evidence of modified contrast at grain boundaries, thus pointing to direct observation of a high-defect tolerance. Furthermore, we show that the low intrinsic bulk conductivity of BiVO_4_ limits electron current through the film, and that at large bias, the transport mechanism can be attributed to a space charge-limited current in the presence of trap states. This study highlights the importance of mechanistic understanding of nanoscale physical interaction to unravel the extraction of charge carriers from heterogeneous photoelectrodes.

## Results

### Nanoscale mapping of photogenerated carriers in BiVO_4_ photoanodes

**Fig. 1:**
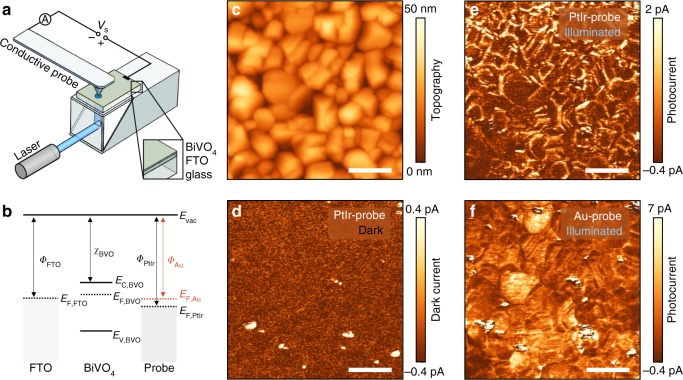
Photoconductive AFM on BiVO_4_ photoanodes. **a** Schematic illustration of the photoconductive atomic force microscope (pc-AFM) setup. The BiVO_4_ film is illuminated from the FTO side through the transparent glass substrate. The bias voltage (*V*_s_) is applied between the metal-coated probe and the transparent FTO back electrode. **b** Schematic illustrations of the relative energetic levels of FTO, BiVO_4_, and metal-coated probes. The solid lines indicate the vacuum level *E*_vac_, and the valence and the conduction band edge of BiVO_4_ (*E*_C,BVO_ and *E*_V,BVO_). The dashed lines show the Fermi level of FTO (*E*_F,FTO_), BiVO_4_ (*E*_F,BVO_), and Au-coated and PtIr-coated probes (*E*_F,Au_, and *E*_F,PtIr_). The arrows indicate the work function of FTO (*Φ*_FTO_), the electron affinity of BiVO_4_ (*χ*_BVO_), and the work function of Au-coated (*Φ*_Au_) and PtIr-coated probes (*Φ*_PtIr_). **c** Topography of BiVO_4_ thin film. **d** Dark current map is acquired with a PtIr-coated probe at an applied bias of 1.75 V. Corresponding photocurrent maps of the same area are acquired with a PtIr-coated probe (**e**) and an Au-coated probe (**f**) at an applied bias of 1.75 V and 1.45 V, respectively. The scale bar is 200 nm

Experiments were performed using spin-coated BiVO_4_ thin films on fluorine-doped tin oxide (FTO)-coated glass substrates (Supplementary Figs. 1 and 2) with photoelectrochemical performance, comparable to the best values reported for undoped BiVO_4_ photoanodes (Supplementary Fig. 3)^19,20^. For pc-AFM measurements, a bias voltage (*V*_s_) was applied to the FTO back electrode and the current was collected by the metal-coated probe at virtual ground (Fig. 1a). The BiVO_4_ samples were illuminated from the FTO side using CW-diode lasers as the light source. Importantly, the choice of the coating material for the probe influences the band alignment at the BiVO_4_/probe interface, thereby allowing investigation of heterogeneous interfacial charge transfer processes, with mechanistic insights provided by varying the nanoscale junction energetics (Fig. 1b).

Figure 1c shows a representative AFM topography image of a polycrystalline BiVO_4_ film with grain sizes between 50–200 nm. Current measurements were performed with a PtIr-coated probe under dark conditions (Fig. 1d) and under illumination (*E*_ph_ = 3.06 eV, Fig. 1e) at an applied sample bias of *V*_s_ = 1.75 V. Without illumination, we observe almost no current contrast, except for small spots (about 20 nm in diameter) that exhibit higher conductivity than the rest of the sample. These conductive spots are shunts between the PtIr-coated probe and the conductive FTO back electrode due to the presence of pinholes within the BiVO_4_ thin films (Supplementary Fig. 4)^20^. These results are in contrast with a previous c-AFM study on epitaxial pulsed laser-deposited BiVO_4_ films (160 nm), where an enhancement of the dark current at grain boundaries was found^30^. However, as noted by Zhang et al.^30^, the grain boundary domains of epitaxial BiVO_4_ are different from grain boundaries of polycrystalline films, as those analyzed in our study.

Under illumination (*E*_ph_ = 3.06 eV, ~100 mW cm^−2^), a clear photoresponse with an average photocurrent of 1.1 pA (*V*_s_ = 1.75 V) and a pronounced heterogeneity across the sample is observed using PtIr-coated probes (Fig. 1e). Notably, control experiments are performed to ensure that the measurements are reproducible and free of topographic artifacts (Supplementary Figs. 5 and 6)^26^. In order to probe the contribution of excitation of trapped charge carriers within the bandgap, we conduct experiments that illuminate the sample using *E*_ph_ = 2.33 eV. However, no detectable photoresponse arises for the sub-bandgap illumination (Supplementary Fig. 7). Therefore, the observed photoconductivity can be unequivocally assigned to the generation of electron-hole pairs across the bandgap. Performing equivalent measurements at the exact same area using an Au-coated probe (Fig. 1f), we observe a photoresponse with a higher average photocurrent of 3.9 pA (*V*_s_ = 1.45 V) and a more spatially homogeneous distribution than with PtIr-coated probes. This pronounced dependence of photocurrent distribution on the probe material suggests that systematic variation of the interfacial energetics of the nanoscale contact can, indeed, be used to probe different properties of the BiVO_4_ photoabsorber, as described below.

**Fig. 2:**
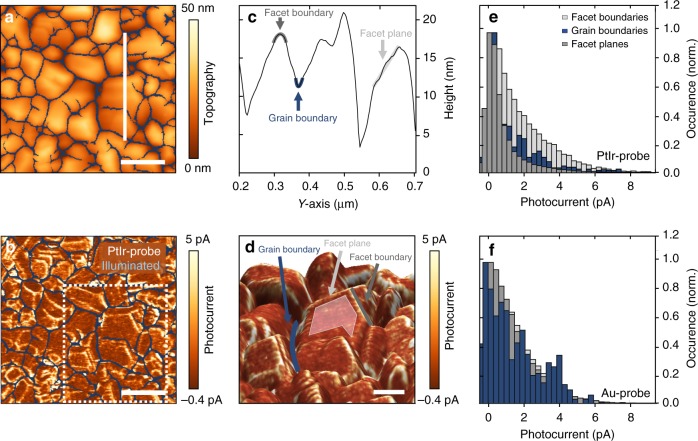
Statistical analysis of the photocurrent distribution. **a** Topography image and **b** photocurrent map of a BiVO_4_ thin film using a PtIr-coated probe at an applied sample bias of 1.75 V. The grain boundaries are highlighted in blue. The spatially resolved photocurrent map is calculated by subtracting the current maps under illumination and under dark conditions. The scale bar is 200 nm. **c** Height profile along the white perpendicular solid line shown in **a** defines facet planes, as well as grain and facet boundaries. **d** 3D-topography image with overlaid photocurrent contrast. A representative facet plane is highlighted in light gray, grain boundary in blue, and facet boundary in dark gray. **e**, **f** Histograms of the photocurrent distributions at facet planes, facet boundaries, and grain boundaries. Histograms shown in **e**, **f** are extracted from **b** and Supplementary Fig. 11d, respectively. Similar distributions are observed in different maps taken in different sample areas. While the current distributions obtained with Au-coated probes show homogenous current distribution across all three regions, the current distributions obtained with PtIr-coated probes exhibit a significantly enhanced photocurrent at facet boundaries

The photocurrent maps obtained with PtIr-coated and Au-coated probes (Fig. 1e, f) show heterogeneity between different grains across the sample. However, no systematic correlation between grain size and average photocurrent per grain is discernible (Supplementary Fig. 8). We statistically analyzed and quantified the photocurrent distribution to determine the correlation between photoresponse and topographic features. For this purpose, we define grain boundaries, plane facets, and facet boundaries as distinct regions in the topography and photocurrent maps (Fig. 2a, b). In the line profile in Fig. 2c, the grain boundaries appear as concave valleys (blue), the facet planes within a single grain are identified by a negligible curvature (light gray), and the facet boundaries are defined as the lines of intersections between facet planes within a single grain, which are characterized by convex curvature in the line profile (dark gray). To provide a better insight into the structural details of the investigated film, all three regions are highlighted in Fig. 2d, which shows an enlarged 3D representation of the topography in Fig. 2a, overlaid with the photocurrent contrast (dotted square in Fig. 2b).

Figure 2e depicts the normalized photocurrent distributions corresponding to facet boundaries and grain boundaries, as well as to facet planes, measured with a PtIr-coated probe. Interestingly, the median photocurrent of 1.31 pA for facet boundaries (dark gray) is significantly higher than the values of 0.51 pA for facet surfaces (light gray) or 0.43 pA for grain boundaries (blue). The higher photocurrent at the facet boundaries is clearly discernible in the overlay image in Fig. 2d. A similar contrast as in the PtIr-photocurrent maps appears in the curvature map calculated across the topography image (Supplementary Fig. 9), thereby supporting the presence of higher current at facet boundaries. The same statistical approach is applied to photocurrent maps recorded with Au-coated probes, and the corresponding histograms are shown in Fig. 2f. In contrast to the results collected with PtIr-coated probes, all three regions exhibit a similar photocurrent distribution. Specifically, facet boundaries and facet planes have a median photocurrent of 1.06 pA and 0.95 pA, respectively, whereas the value for grain boundaries of 0.75 pA is only slightly lower. Interestingly, while slow carrier transport has already been pointed out as a major shortcoming of this material^16^, the photocurrent values in the proximity of grain boundaries suggest that they neither act as recombination centers nor do they generate a potential barrier for majority carriers in BiVO_4_, as opposed to hematite^31^, ZnO platelets^32^, or CdTe/CdS thin films^29^. These findings highlight that internal interfaces are defect tolerant, thus explaining how nanostructuring approaches based on BiVO_4_ yield highly efficient photoanodes^8^.

### Analysis of charge carrier transport mechanism

While photocurrent maps detail heterogeneities of the optoelectronic properties on a nanometer scale, they provide only limited information about the underlying charge-transport mechanism. To elucidate the conduction mechanism, single point high resolution current-voltage curves were recorded on plane facets at a fixed sample position. For this analysis, we first determined the work functions of different materials (Supplementary Table 1) and estimated the barrier height, which is ideally derived by the difference between the work function of the probe and the electron affinity of BiVO_4_. Based on the work functions of the probe materials, we expect that the Schottky barrier is reduced by approximately 0.1 eV for Au-coated compared to PtIr-coated probes.

**Fig. 3:**
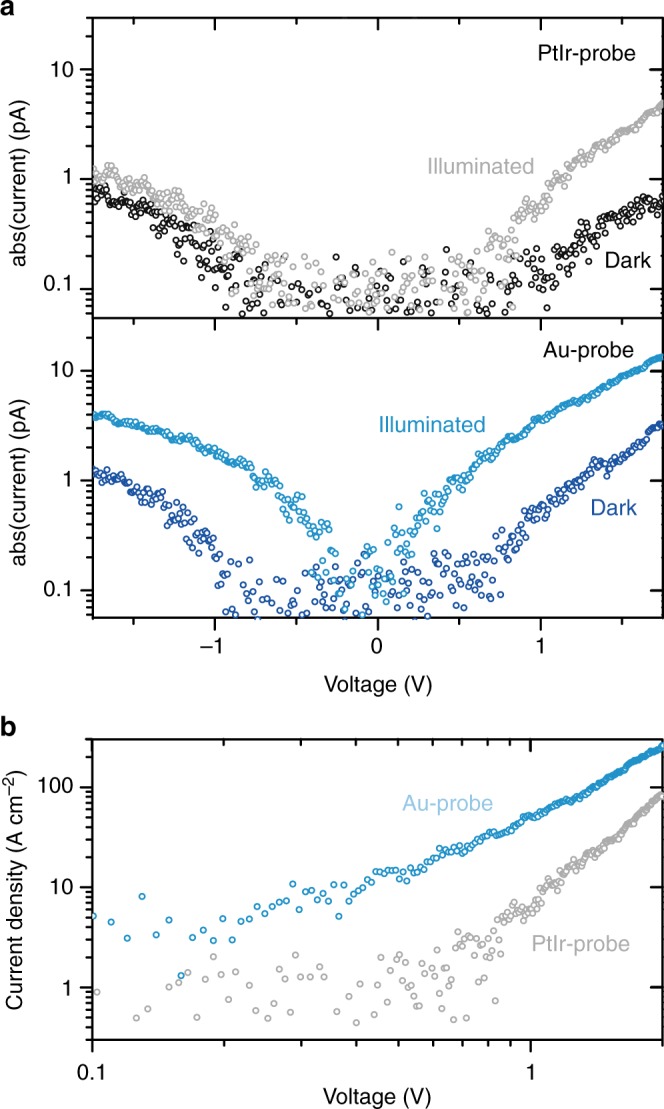
Dark and photocurrent characteristics. **a** *IV*-characteristic of the FTO/BiVO_4_/probe circuit under illumination and in dark for PtIr-coated and Au-coated probes at the same sample position. **b** Current density under illumination for PtIr-coated and Au-coated probe calculated using the corresponding contact area between probe and BiVO_4_. We used the Derjaguin–Mueller–Toporov continuum mechanical model to estimate the contact area between the surface and the probe^34^. All *IV*-curves show strong nonlinear behavior, thereby excluding ohmic contact and wiring resistance as limiting factors in overall electronic transport through the photoanode device

For PtIr-coated and Au-coated probes, Fig. 3a shows representative *IV*-curves of the FTO/BiVO_4_/probe circuit in the dark (black and dark blue circles) and under illumination (gray and light blue circles). The *IV*-curves for forward (*V*_s_ < 0) and reverse bias (*V*_s_ > 0) are almost symmetric and deviate from the conventional rectifying behavior of Schottky contacts (Supplementary Fig. 10). The enhanced current in the reverse bias regime is in agreement with the behavior of nanoscale Schottky junctions, which usually show smaller current under forward bias and inverse rectification under reverse bias^33^.

In the following section, we focus on the positive bias regime, which is analogous to the operating conditions of BiVO_4_ photoanodes. Higher dark and photocurrents are reproducibly observed with Au-coated probes, compared to PtIr-coated probes on different BiVO_4_ samples and probe batches (Supplementary Fig. 11). Interestingly, in a nanoscale Schottky contact, the depletion width is reduced with decreasing the tip radius *r*. This effect leads to an increased tunneling probability at the interface in the reverse low-bias regime and the expectation of a higher current for smaller tip radius, which is in contrast with the observed higher absolute current in the case of Au-coated probes (*r* *<* 35 nm radius of curvature), with respect to the PtIt-coated probes (*r* *<* 25 nm radius of curvature)^33^. However, the tip radius of the curvature does not describe the actual probe/sample contact area during measurement.

Therefore, to account for different contact areas and to enable quantitative comparison, we show the calculated current density obtained via the Derjaguin–Mueller–Toporov continuum mechanical model^34^, in Fig. 3b. The calculated contact area for PtIr-coated probes (28 nm^2^) is about three-times larger compared to Au-coated probes (8 nm²). Interestingly, the larger contact area should lead to higher current values for the PtIr-coated compared to the Au-coated probe. However, the opposite is observed, which indicates that energetics of these nano-Schottky junctions plays a key role in defining the transport properties, as described below.

**Fig. 4:**
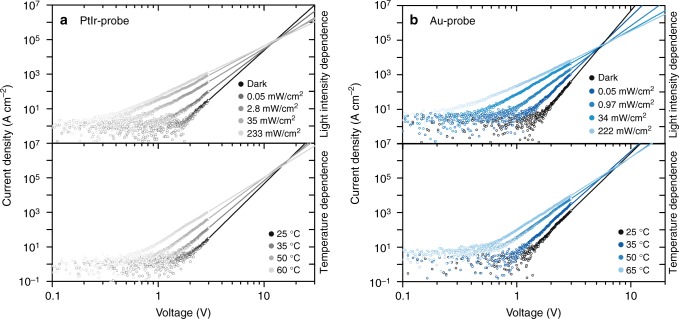
Charge transport mechanism in BiVO_4_. Temperature and light intensity-dependent *JV*-characteristics of the FTO/BiVO_4_/probe circuit for PtIr-coated probes (**a**) and Au-coated probes (**b**). For high-bias voltages *V*_s_ > 0.7 V, the curves show a linear relationship of log(*J*) and log(*V*), which corresponds to a power law dependence *J *∝ *V*^*m*^. Solid lines are power law fits to the data in the high-bias regime. The extrapolated *JV*-characteristics for different intensities (temperatures) intersect at the crossover voltage *V*_c_. The power law dependence and the existence of a crossover voltage are signatures of space charge-limited conduction in the BiVO_4_ film

Shedding light on the type of limiting-transport mechanism can help understand the nanoscale heterogeneity in BiVO_4_ thin films and provide new insights into its macroscale behavior. Therefore, we analyze the *IV*-characteristics for the high-bias regime *V*_s_ > 0.7 V, at which the pc-AFM maps were recorded. Notably, at low-bias voltages *V*_s_ < 0.7 V, the measured current is very low and hardly discernible from the noise level. *IV*-characteristics were measured using PtIr-coated and Au-coated probes as a function of light intensity and temperature. When increasing the temperature or light intensity, we observe an increase in photocurrent, a shift of the threshold voltage at which the current increases, and smaller slopes of the *IV*-characteristics for both probe coatings (Fig. 4). While the nano-Schottky contact might influence the early current onset in the low-bias regime, the observed temperature dependence of the dark current excludes the interface-limited current due to Fowler–Nordheim injection, which is a temperature independent tunneling process^35^. For bulk-limited transport, Poole–Frenkel emission is expected to yield a linear relationship between ln(*J*/*V*) and *V*^0.5^, with a slope of *β*/(*k*_B_*T*) with $\beta = \sqrt {e/(\pi {\it{\epsilon }}_0{\it{\epsilon }}_{\mathrm{r}})}$ (Supplementary Table 2). Under dark conditions, the slope is 0.018 for Au-coated and 0.014 for PtIr-coated probes, respectively. Both values are significantly higher than the theoretically calculated slope of 0.0041, assuming ${\it{\epsilon }}_{\mathrm{r}} = 52$, indicating that Poole–Frenkel conduction is unlikely to be the dominant conduction mechanism. We can also rule out the hopping conduction, since we calculate a hopping distance of about 2–3 nm for both Au-coated and PtIr-coated probes in the high-bias regime, which is significantly higher than the previously reported values of about 0.4 nm for BiVO_4_ single crystals^17,36^.

Overall, we find that the asymptotic behavior of the *JV*-charactersitics is best described by a linear relation between log(*J*) and log(*V*) (Fig. 4), which implies a power law dependence, and thereby a space charge-limited current (SCLC). The solid lines represent the fitting of the high bias regime according to *J *∝ *V*^*m*^. The corresponding power law exponent is *m* = 6 under dark conditions and room temperature, and it decreases for increasing light intensity to *m* = 2.8 (2.9) and for increasing temperature to *m* *=* 4.2 (3.8) for Au-coated (PtIr-coated) probes. Notably, the extrapolated fit curves exhibit a characteristic crossover voltage *V*_c_. Such a power law dependence and the existence of a crossover voltage are strong indications for SCLC^37–40^. However, a power law with a characteristic exponent of *m* *=* 2 describes the SCLC in ideal trap-free materials. In the presence of trap states (shallow or deep), the SCLC follows a modified power law with *m* *>* 2. In the SCLC model with trap states, the slope *m* converges towards *m* = 2 with increasing light intensity or sample temperature^37,41^, and the extrapolated *JV*-curves intersect at the characteristic crossover voltage *V*_c_, which agrees with our observations. The slope of the *JV*-curves in a log-log plot is determined by 1+*E*_t_/(*k*_B_*T*), whereas the crossover voltage *V*_c_ is directly related to the trap density $H_{\mathrm{t}} = \frac{{2V_{\mathrm{c}}{\it{\epsilon }}_0{\it{\epsilon }}_{\mathrm{r}}}}{{qd^2}}$, which can be calculated for a known film thickness *d* and permittivity ${\it{\epsilon }}_{\mathrm{r}}$^37,38,42,43^. Under these assumptions, from the crossover voltages *V*_c_ for the Au-coated and PtIr-coated probes, we can estimate the trap density *H*_t_ to be between 1.0 × 10^19^ cm^−3^ and 2.2 × 10^19^ cm^−3^ assuming a film thickness of 60 nm and dielectric constant for BiVO_4_ of ${\it{\epsilon }}_{\mathrm{r}} = 52$. These values are about two order of magnitudes higher compared to reported trap densities for TiO_2_ thin films^44^ and organic^45^ solar cells. Furthermore, we calculated the characteristic energy of the traps and obtained *E*_t_ = (0.11 ± 0.02) eV for both the Au-coated and the PtIr-coated probes, thereby indicating shallow trap levels. Under illumination, the characteristic energy of the traps is reduced to about *E*_t_ = (50 ± 5) meV, which indicates trap state filling by photogenerated charge carriers. Shallow defect levels have been reported for donor-like oxygen vacancies, whereas vanadium interstitial or vanadium on bismuth sites are associated with deep donor levels^46^. Interestingly, the characteristic energy of traps is in the same range as the energy barrier associated with carrier generation by ionization of electrons from donor states to small-polaron transport states, which is about 50 meV^36^.

Interestingly, the analysis of the temperature and illumination intensity dependent different *IV*-curves reveals a similar transport mechanism using PtIr-coated and Au-coated probes, whereas different heterogeneities are observed in the corresponding photocurrent maps. The observed heterogeneity is reflected by representative *IV*-curves extracted at grain boundaries, facet boundaries, and facet planes from voltage-dependent current maps. For PtIr-coated probes, the *IV*-curves of facet boundaries are shifted towards smaller voltages with respect to the *IV*-curves of grain boundaries and facet planes (Supplementary Fig. 12a). By contrast, for Au-coated probes, all areas exhibit similar *IV*-curves (Supplementary Fig. 12b), thus resulting in a more homogeneous contrast. It is worth noting that while we can acquire single point *IV*-curves at high applied forces, continuous mapping at high forces results in sample damage (Supplementary Figs. 13 and 14). Therefore, to specifically investigate the mechanical sample/probe contact, we acquired force dependent *IV*-curves and current maps for both probe coatings. In this context, force dependent analysis shows that the sample/probe contact can be improved by increasing the applied forces (Supplementary Fig. 15). We observe that PtIr-coated probes show a more pronounced force dependence of the current (Supplementary Fig. 13f) with respect to the Au-coated probes (Supplementary Fig. 14f). These findings suggest that, for Au-coated probes, a good electric contact is already established at low forces, which has been previously observed in ultra-low force contacts^47,48^. Therefore, we hypothesize that the origin of the different photocurrent heterogeneities observed with PtIr-coated and Au-coated probes may be related to the contact at the BiVO_4_/probe interface. To further confirm our hypothesis that the contact at the sample/probe interface determines the contrast in current mapping, additional pc-AFM measurements were conducted using TiN-coated probes. The TiN-coated probes have a tip radius (*r* *<* 20 nm) and a hardness comparable to PtIr, and a low work function of 4.7 eV comparable to Au. Consequently, current maps with TiN-coated probes show an intermediate image contrast between Au and PtIr-maps (Supplementary Fig. 16). Compared to Au-coated probes, the current distribution appears more heterogeneous with a decreased current at facet planes, and an increased current at facet boundaries (Supplementary Fig. 17). These results supports that the contrast in heterogeneity within the same maps is also governed by the BiVO_4_/probe interface. Additionally, we consider that the current contrast might be influenced by geometric field enhancement between the probe and sharp topographic features, such as the facet boundaries, which can lead to an amplified current in these specific regions^49,50^.

## Discussion

Previous studies demonstrated that small polaron hopping is the dominant electron transport mechanism in BiVO_4_ for temperatures between 250 K and 400 K, yielding a very low electron mobility of about 4 × 10^−2^ cm^2^ V^−1^ s^−1^ ^16,17,36^. For adiabatic polaron hopping, Emin et al. derived an expression for mobility that takes into account the effect of the electric field on the hopping rate and has the same functional dependence as the Poole–Frenkel effect^51^. However, as discussed above, the observed slope of the *JV*-curves does not support the Poole–Frenkel effect. Therefore, we rule out the electric field enhancement of the polaron mobility as the dominant non-linear contribution to the conductance in the high-field regime. The SCLC model suites well materials with low bulk conductivities and charge carrier mobilities, and it has been consistently used with organic small molecules and polymers. Similarly, we note that transport in organic semiconductor single crystals has been recently described by a SCLC modified with a field-dependent small polaron mobility^52^. Although such a minor correction might be applicable for the case of BiVO_4_ thin films, the strong illumination density dependence of the slope of the *JV*-curves indicates that a SCLC in the presence of trap states is the dominant mechanism in our samples.

In conclusion, we measured photocurrent maps and single-point *IV*-curves in BiVO_4_ photoanodes by pc-AFM, revealing the morphology-dependent nanoscale heterogeneity. We utilized PtIr-coated and Au-coated probes with different work functions and mechanical properties. We demonstrate that the choice of the conductive probe material and the resulting contact at the sample/probe interface can influence the image contrast in (photo)conductive mapping. In addition, we find that BiVO_4_ shows a high defect tolerance with no additional recombination sites at grain boundaries, thereby explaining how nanostructuring approaches and polycrystalline films exhibit high efficiency in the presence of internal interfaces. This study proves that the current is bulk limited, and largely dominated by the space charge-limited current in the presence of trap states. Since trap states are the limiting factor for the current through the BiVO_4_ film, their presence will have a significant impact on macroscale photocurrent generation and can affect charge extraction at high overpotential. Our results emphasize the importance of carefully considering contact formation between the nanoscale conductive probe and low conductivity metal-oxide semiconductors such as BiVO_4_ for accurate characterization of charge transport in these materials. Ultimately, these considerations are highly relevant for operation of light absorbers in catalytic reactions, and support the importance of reducing bulk recombination due to trap states through nanostructuring approaches for improving the performance of BiVO_4_. The accurate characterization of the charge-transport mechanism is necessary to unravel the performance limitations in energy materials, and to eventually enable design and development of new functional systems.

## Methods

### Sample preparation

All measurements were conducted on spin-coated bismuth vanadate thin films deposited on FTO/glass substrates^20^. Prior to deposition, the FTO/glass slides were thoroughly cleaned with isopropanol, detergent in deionized water, and pure deionized water. Subsequently, the samples were dried with nitrogen and treated for 10 min in a UV-ozone cleaner (Jelight Model 42). For spin-coating of BiVO_4_, 15 mL of a 0.2 M solution of bismuth (III) nitrate pentahydrate (Sigma Aldrich, ≥98%) in acetylacetone (Sigma Aldrich, ≥99%) and 100 mL of a 0.03 M solution of vanadium(IV)-oxy acetylacetonate in acetylacetone were prepared. The two solutions were first sonicated separately for 10 min. Afterwards, both were mixed together and sonicated for an additional 5 min. For a homogenous BiVO_4_ layer, 1–1.2 mL of the resulting solution was filtered with 0.45 μm nylon filters (Thermo Scientific) and dispensed onto the freshly cleaned FTO/glass substrate. The substrate was then spun twice at 1000 r.p.m. for 6 s with an acceleration rate of 150 r.p.m./s. Subsequent to the spin-coating cycle, the substrate was annealed in a muffle furnace for 10 min at 500 °C. The spin-coating/short annealing cycle was repeated nine times to achieve a final thickness of ~50 nm. After the final spin-coating cycle, the substrate was annealed for 2 h at 500 °C.

The presence of the photoactive monoclinic scheelite phase and the phase purity is verified by grazing incident X-ray diffraction and Raman spectroscopy (Supplementary Fig. 2). Macroscopic photoelectrochemical performance of these films, evaluated by cyclic voltammetry in the presence of a hole scavenger (0.1 M Na_2_SO_3_) at near neutral pH (1 M KPi buffer) show a photocurrent density of 2.0 mA cm^−2^ and 1.8 mA cm^−2^ at 1.23 V, against a reversible hydrogen electrode for back- and front-side illumination (AM 1.5 standard spectrum, 100 mW cm^−2^), respectively (Supplementary Fig. 3). The slight difference in performance between the back and front illumination is ascribed to the transport properties of charge carriers. Specifically, while photogenerated holes can effectively migrate to the surface (hole diffusion length of 70–100 nm)^16,17^, BiVO_4_ films show bulk electron transport limited by the small mobilities of small polarons^9^. This photoelectrochemical performance is in agreement with the best value obtained for undoped BiVO_4_ thin films, yet far from the theoretical value of 6.25 mA cm^−2^ for a 2.5 eV bandgap material.

### Photoconductive AFM and KPFM

All pc-AFM mapping and *IV*-curves measurements were performed with a commercial AFM system (Bruker Dimension Icon). For pc-AFM mapping, PeakForce TUNA mode was used. In PeakForce mode, a force–distance curve is recorded at each pixel of an image, from which the measured quantities are extracted, allowing simultaneous investigation of surface morphology, (photo)conductivity, and material properties such as adhesion force or deformation. Furthermore, this technique is extremely gentle and reproducible, since the applied maximum contact force is known, set at a minimum value, and kept constant throughout the measurement. During the lateral movement, the cantilever is not in contact with the surface, which reduces friction, lateral forces, and surface or tip damage, thereby minimizing possible artifacts. For the photocurrent maps, the sample was illuminated from the back using a specially designed illumination setup. The light intensity was varied between 0 and 250 mW cm^–2^. As light sources, laser diodes with wavelengths of 405 nm and 532 nm were used. For all pc-AFM measurement, cantilevers with a nominal spring constant of 2.8 N m^−1^ or 0.5 N m^−1^ and a conductive metal coating of PtIr (Bruker SCM-PIT) or Au (MikroMasch HQ:NSC19/Cr-Au) were used. Alternatively, Si cantilevers (nominal spring constant of 2.8 N m^−1^) were coated by atomic layer deposition with a 10 nm thick conductive TiN layer. All scan parameters for each type of probe coating were optimized with respect to good signal-to-noise ratio without damaging the BiVO_4_ sample. *IV*-curves were recorded by sweeping the bias from negative to positive values. At positive-bias voltage, electrons are injected from the probe into the BiVO_4_ thin film and are collected by the FTO back contact.

The work functions of the probe materials were experimentally determined by means of Kelvin probe force microscopy (KPFM), using freshly cleaved highly oriented pyrolytic graphite (HOPG) as the reference. Subsequently, the calibrated cantilevers were used to measure the work function of BiVO_4_ in a similar way (Supplementary Table 1).

### Data availability

The source data that support the findings of this study are included in the Article, Supplementary Information, and Supplementary Data 1.

## Electronic supplementary material

Supplementary Information

Description of Additional Supplementary Files

Supplementary Data 1
