# Supplementary material for: Nanoscale imaging of charge carrier transport in water splitting photoanodes
Source: Nat Commun. 2018 Jul 16;9:2597. doi: 10.1038/s41467-018-04856-8 (PMC6048052; doi:10.1038/s41467-018-04856-8)
Supplement: Supplementary file 1 — Supplementary Information [file 41467_2018_4856_MOESM1_ESM.pdf]

Supplementary Information

Nanoscale imaging of charge carrier transport in water splitting photoanodes

Eichhorn et al.

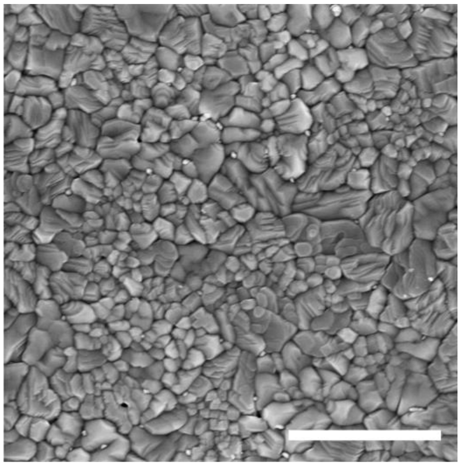

Supplementary Figure 1 Scanning electron microscope image of a $BiVO_4$ thin film. The scale bar is 1 µm.

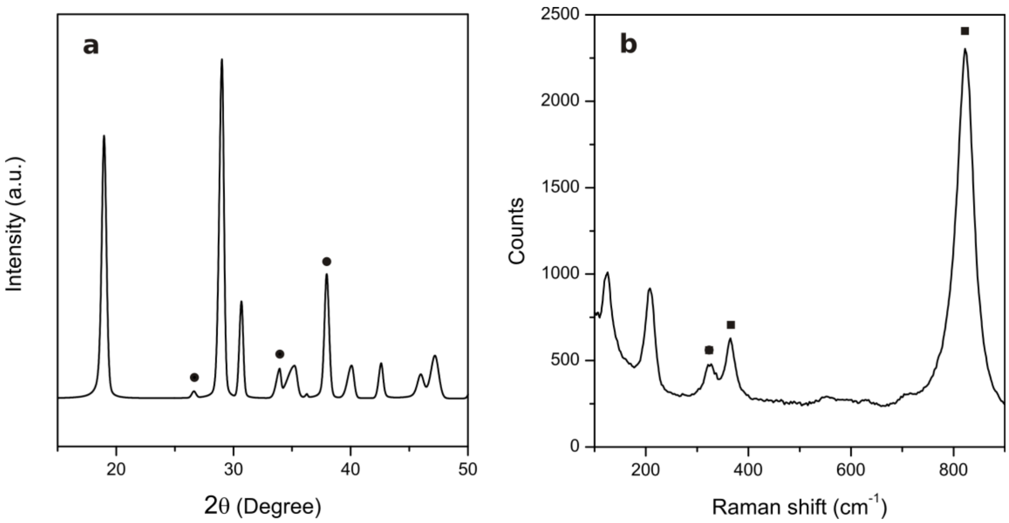

Supplementary Figure 2 XRD (**a**) and Raman spectra (**b**) of spin-coated $BiVO_4$ thin film grown on FTO glass substrate. **a** The FTO peaks are marked with black circles. All other peaks arise from the $BiVO_4$ thin film confirming the monoclinic scheelite structure. **b** The Raman spectrum of monoclinic $BiVO_4$ shows pronounced bands at 828 cm^{-1}, 365 cm^{-1} and 333 cm^{-1} highlighted with black squares.

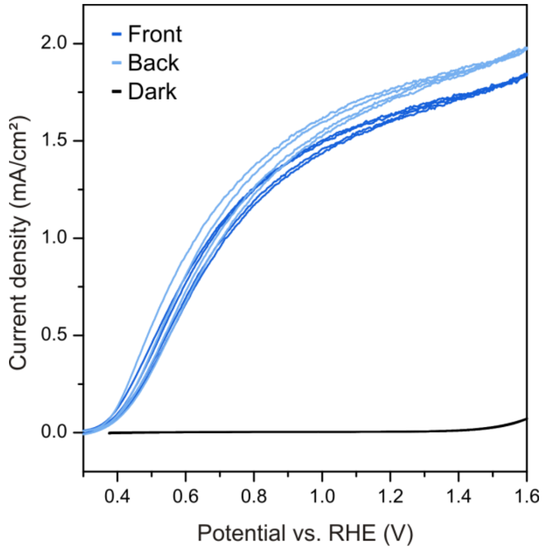

Supplementary Figure 3 Cyclic voltammograms of $BiVO_4$ in 1 M phosphate buffer (pH 6.8) and 0.1 M Na_2SO_3 for frontside (dark blue) illumination, backside (bright blue) illumination, and in the dark (black).

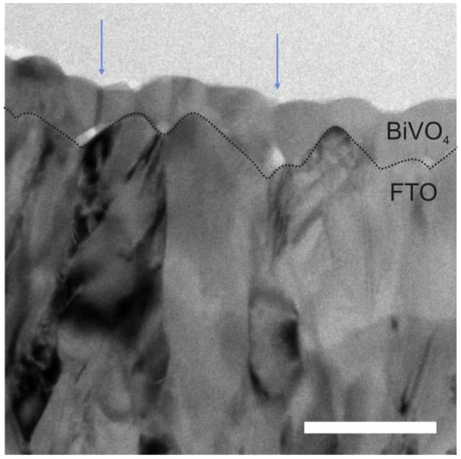

Supplementary Figure 4 TEM cross-section images of pristine $BiVO_4$ thin films. The interface between the FTO and the $BiVO_4$ layer is highlighted by the black dotted line. The blue arrows mark possible starting points for shunts between the FTO and the metal-coated probe. The scale bar is 200 nm. (Reprinted with permission from Ref. [1] Copyright 2016 Nature Publishing Group)

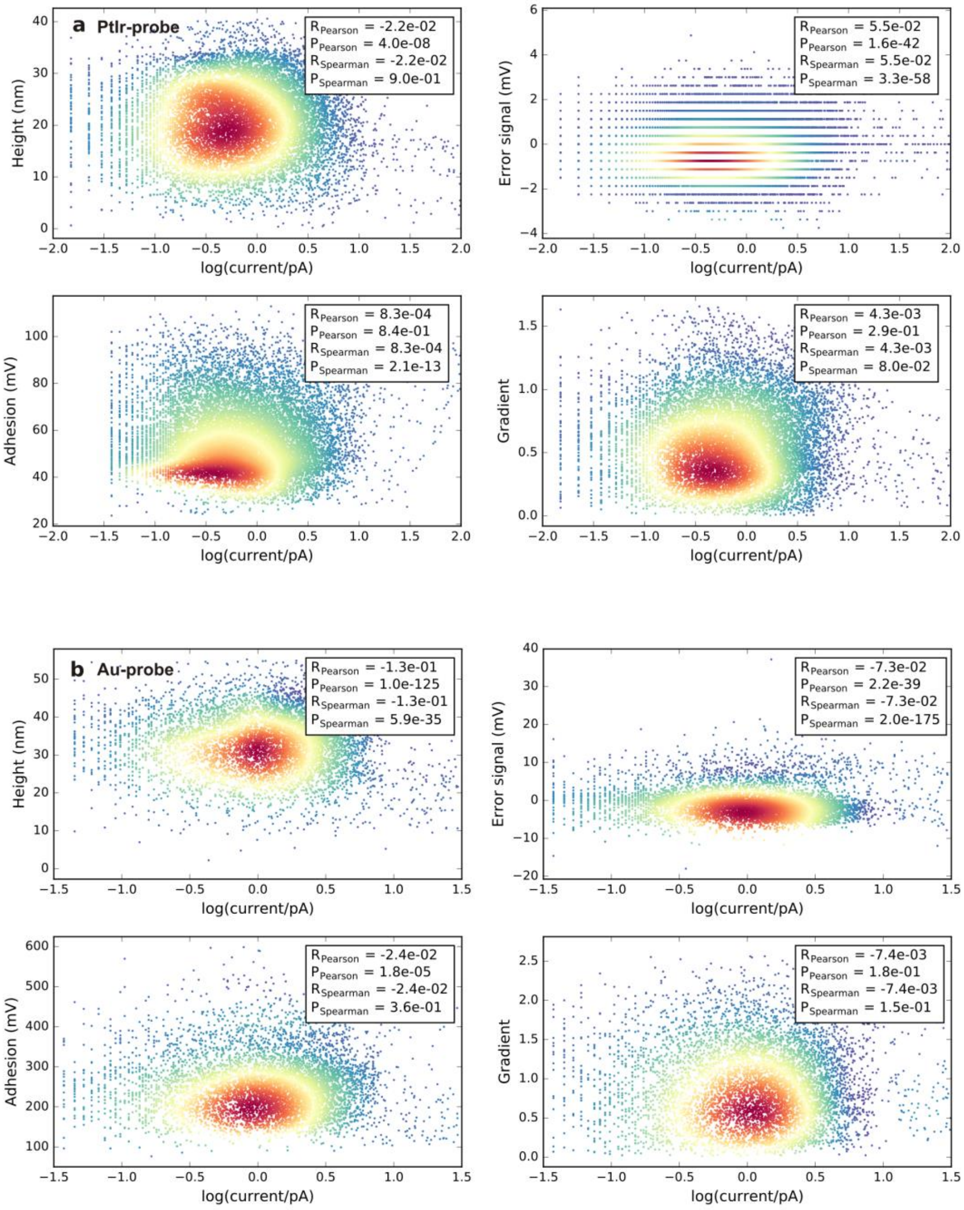

Supplementary Figure 5 Scatter plots of photocurrent measurements and simultaneously measured height, error signal, adhesion, and calculated gradient using (**a**) PtIr-, and (**b**) Au-coated probes. Each point in the plot represents a single pixel within the photocurrent map (x-axis) and the measured property map (y-axis). The color scale indicates the density of data points. For the photocurrent a log-scale was used effectively yielding a normal distribution of values, which is suitable for a correlation analysis. The Pearson coefficient are used to test linear correlation between normally distributed variables. The Spearman coefficient does not require normally distributed variables, and it indicates a monotonic functional relation. The corresponding R-values describe strength and direction of the correlation and they are between -1 and +1. An absolute value close to 1 indicates significant correlation between the variables. The corresponding P-values determine whether the correlation between the variables is statistically significant. A low P-value is taken as evidence that the correlation coefficients are significant. For higher P-values the evidence about the significance of the association between the variables is inconclusive. The corresponding R and P value for Pearson and Spearman correlation are shown in the inset of each plot. All R values are close to zero ($R < 0.055$), which indicates that there is little to no correlation between photocurrent and the measured properties. In turn, the photocurrent shows little to no scan artifacts.

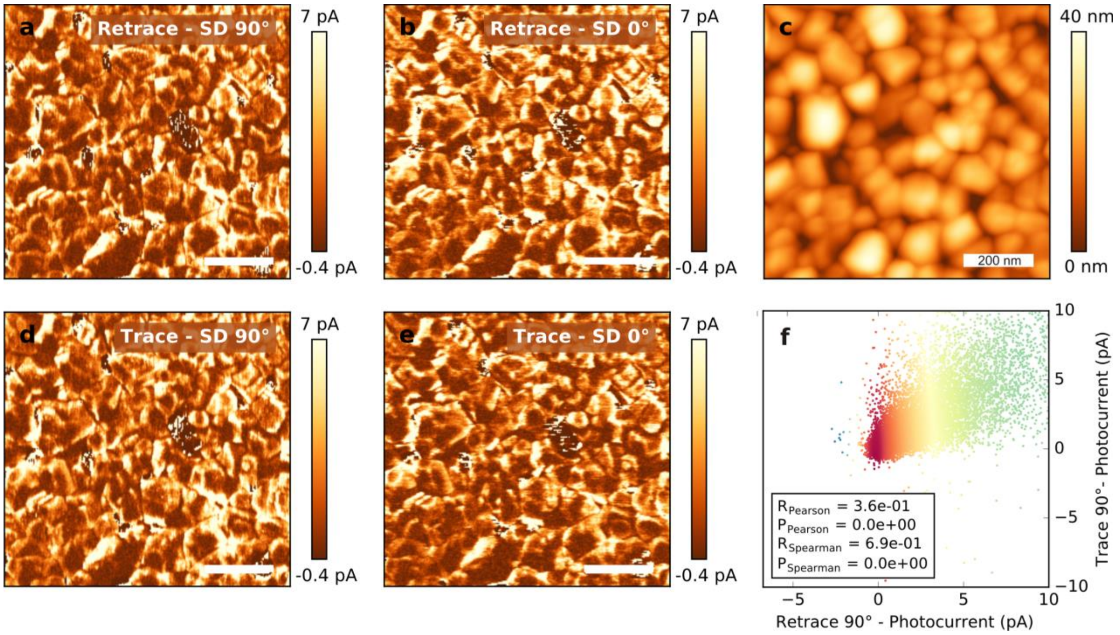

Supplementary Figure 6 Retrace (**a, b**) and trace (**d, e**) photocurrent maps of $BiVO_4$ thin films for 90° and 0° scan direction. **c** Corresponding topography image. **f** Scatter plot of trace and retrace image at a scan angle of 90°. The color scale indicates the density of data points. Pearson and Spearman correlation coefficients are given in the inset. The deviation from ideal correlation arises mainly from of the finite noise level of the current measurement (about 1.5 pA) and sample drift. The scale bar is 200 nm.

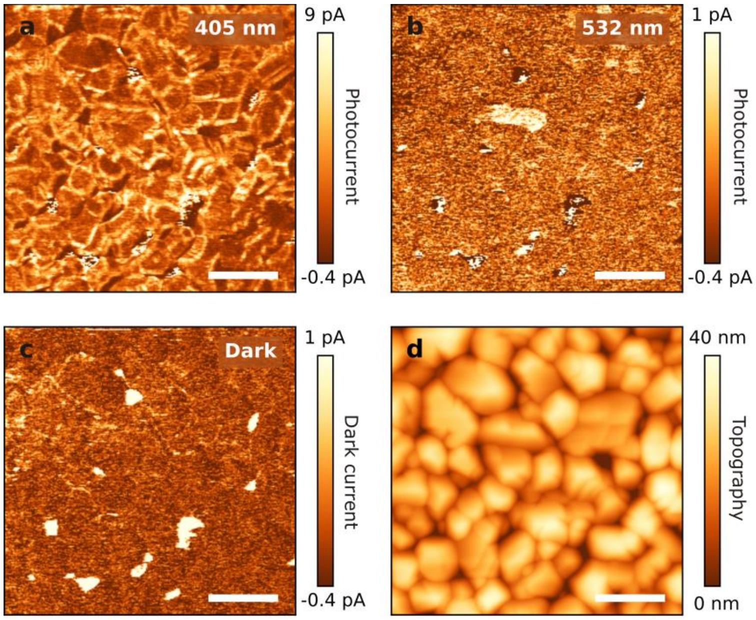

Supplementary Figure 7 Photocurrent image of $BiVO_4$ thin film using a PtIr-coated probe and an applied bias of 1.75 V. The sample is illuminated with 405 nm for above band-gap illumination (**a**) and with 532 nm for sub-band-gap excitation (**b**). **c** The corresponding dark current map, and **d** the topography image at the same sample position. The scale bar is 200 nm.

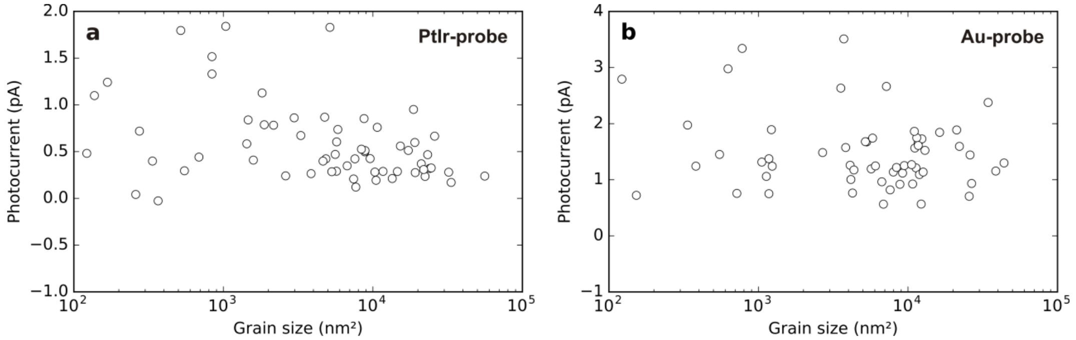

Supplementary Figure 8 Average photocurrent across single grains in dependence of the grain size for PtIr- (**a**) and Au-coated (**b**) probes.

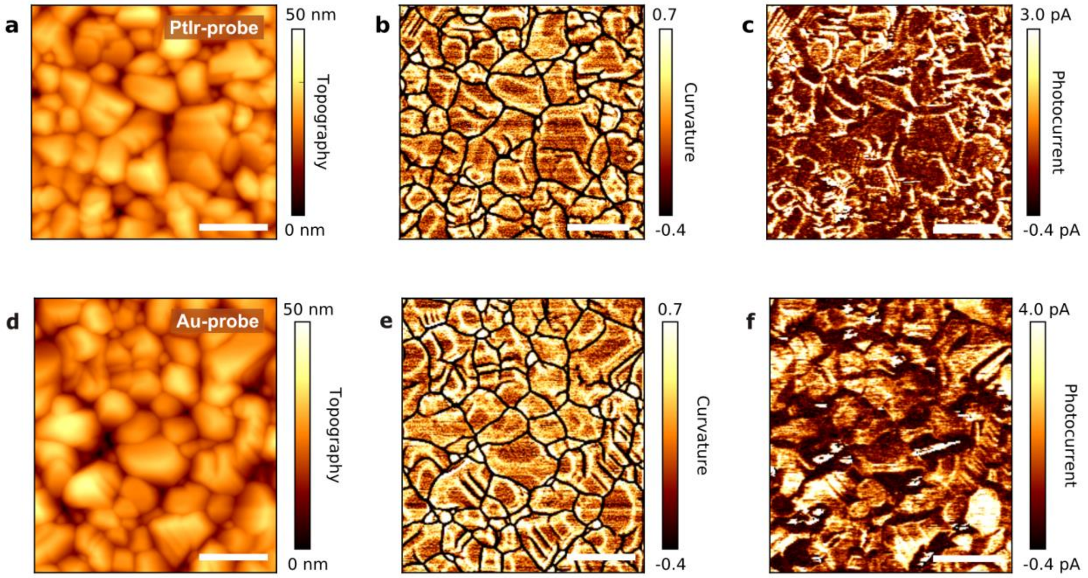

Supplementary Figure 9 Topography map of $BiVO_4$ thin film using a PtIr-coated probe (**a**) and Au-coated probe (**d**). **b**,**e** Curvature map calculated from **a** and **d.** For PtIr-coated probes the curvature map shows the same contrast as the corresponding photocurrent map at 1.75 V applied bias in **c**. In contrast, the photocurrent map for Au-coated probes (**f**) at 1.45 V clearly differs from the curvature map in**e**. The scale bar is 200 nm.

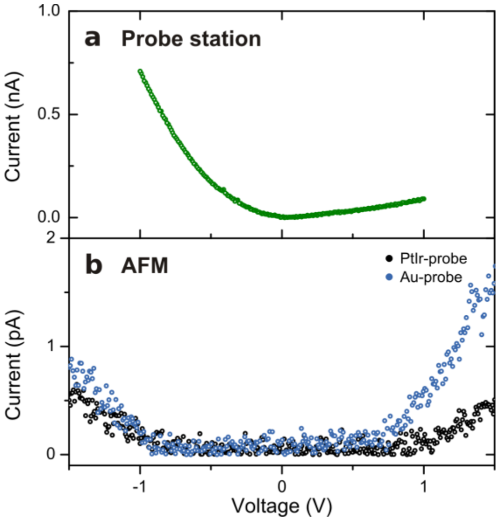

Supplementary Figure 10 *IV*-curves of $BiVO_4$ thin films measured with a probe station (**a**) and conductive AFM (**b**). In both setups the voltage polarity is applied to the sample ensuring the same voltage polarity.

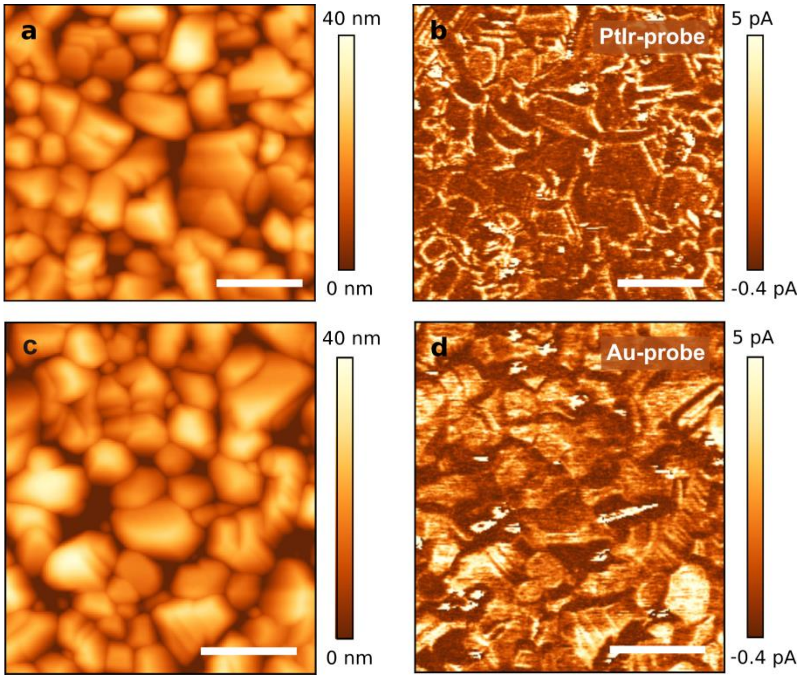

Supplementary Figure 11 Topography (**a, c**) and photocurrent maps (**b, d**) of $BiVO_4$ thin film using a PtIr- and Au-coated probes and an applied bias of 1.75 V and 1.45 V, respectively. The scale bar is 200 nm.

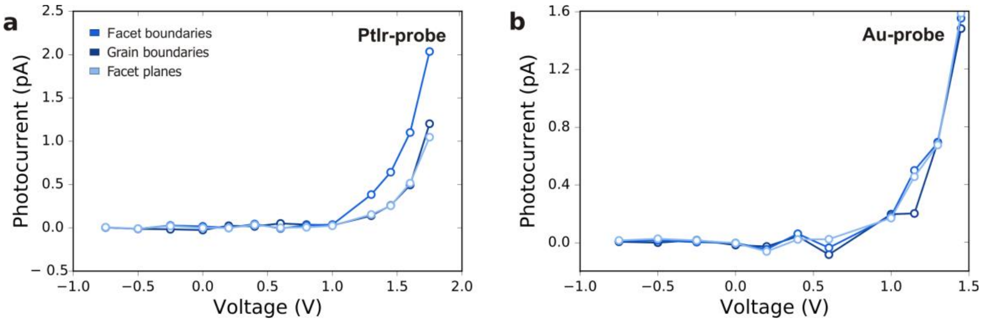

Supplementary Figure 12 *IV*-curves extracted from *IV*-maps averaged over selective morphologies: facet planes, facet boundaries, and grain boundaries for PtIr- (**a)** and Au-coated probes (**b**).

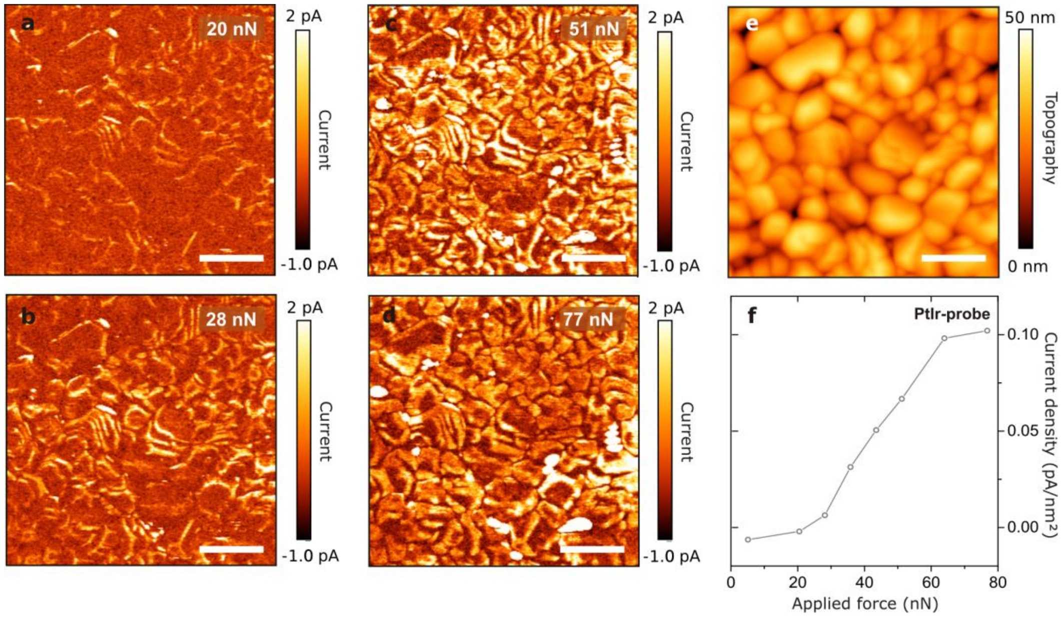

Supplementary Figure 13 Current maps of $BiVO_4$ thin films under illumination using PtIr-coated probes and a constant applied sample bias 1.75 V. The applied force is 20 nN (**a**), 28 nN (**b**), 51 nN (**c**), and 77 nN (**d**). **e** Corresponding topography image at the sample position. The scale bar is 200 nm. **f** Average current density for each current map in dependence of the applied force for PtIr-coated probes.

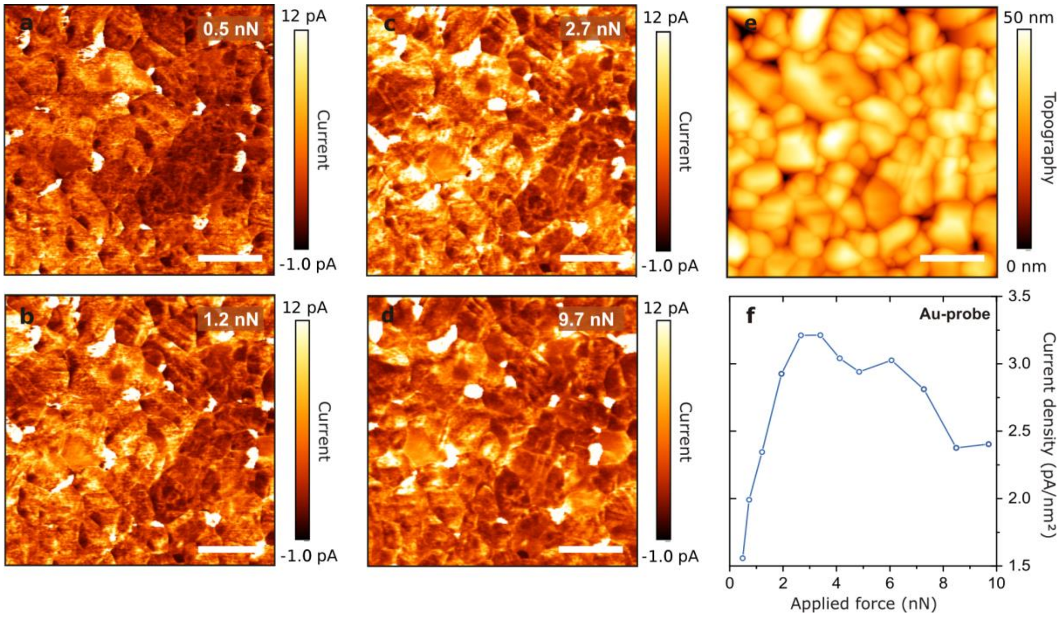

Supplementary Figure 14 Current maps of $BiVO_4$ thin films under illumination using Au-coated probes and a constant applied sample bias 1.45 V. The applied force is 0.5 nN (**a**), 1.2 nN (**b**), 2.7 nN (**c**), and 9.7 nN (**d**). **e** Corresponding topography image at the sample position. The scale bar is 200 nm. **f** Average current density for each current map in dependence of the applied force for Au-coated probes.

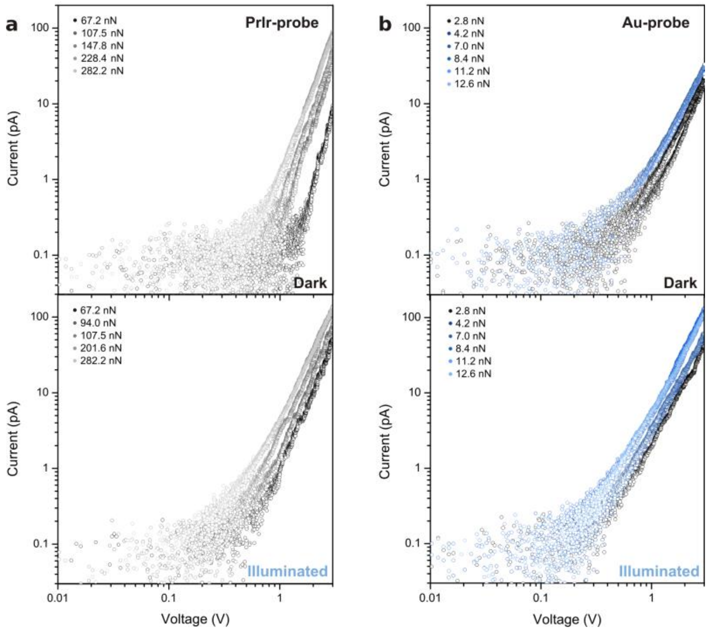

Supplementary Figure 15 *IV*-curves for PtIr- (**a**) and Au-coated (**b**) probes in dependence of the applied force. The *IV*-curves are recorded in the dark and under illumination.

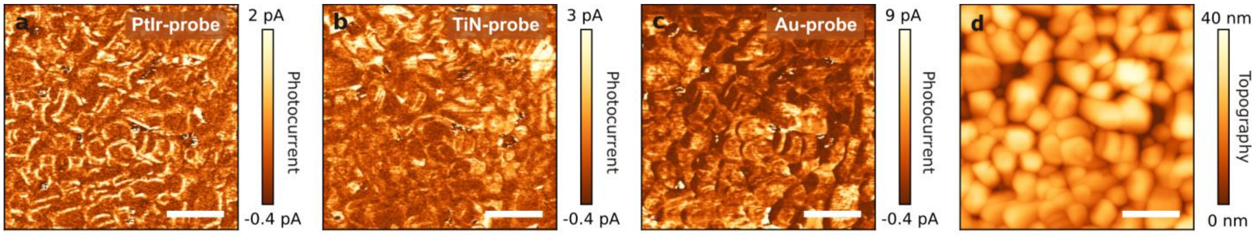

5

Supplementary Figure 16 Photocurrent maps recorded at an identical position on the same sample using PtIr- (**a**), TiN- (**b**), and Au-coated (**c**) probes. The probe coating clearly influences the charge extraction resulting in different photocurrent contrasts. **c** Topography image of the corresponding sample area. The scale bar is 200 nm.

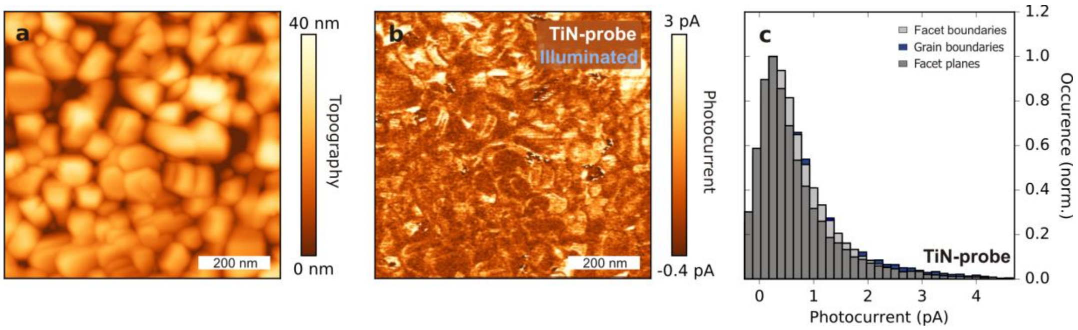

Supplementary Figure 17 Topography image (**a**) and photocurrent map (**b**) of a $BiVO_4$ thin film using a TiN-coated probe and an applied sample bias of 1.75 V. The scale bar is 200 nm. **c** Histograms of the photocurrent distributions at facet planes, facet boundaries, and grain boundaries.

Supplementary Table 1 Work functions of FTO, $BiVO_4$, and different probe coatings. The work function values are experimentally determined by means of Kelvin probe force microscopy.

**Material**	**Work function (eV)**	
	Literature	Experiment
FTO	4.4 - 5.0[2,3]	~ 4.8
$BiVO_4$	4.7-5.3[4,5]	~ 4.7
PtIr	4.3 - 5.5[6-8]	~ 4.9
Au	5.0 - 5.1[7]	~ 4.8
TiN	4.7- 5.0[9,10]	~ 4.7

Supplementary Table 2 Summary of different current conduction models with current density J, electric field E, effective mass m^*, electron charge q, barrier height ϕ, reduced Planck constant $\hbar$, temperature T, Boltzmann constant k_B, ideality factor η, $\beta = \sqrt{q/(\pi\epsilon_0\epsilon_r)}$, mean hopping distance a, characteristic energy of the trap state distribution E_t, density of trap states H_t, permittivity of free space ε_0, dielectric constant of the material ε_r, film thickness d.

Conduction model	Functional dependence of *JV*-curve
Fowler-Nordheim tunneling	$J \propto E^2\, exp\left(-\frac{4\sqrt{2m^*}\,(q\phi)^{3/2}}{3q\hbar E}\right)$
Schottky emission	$J \propto T^2 exp\left(-\frac{q\phi - \frac{1}{2}\beta\sqrt{E}}{k_B T}\right)$
Hopping conduction	$J \propto exp\left(\frac{qaE - \phi}{k_B T}\right)$
Poole-Frenkel emission	$J \propto E\, \exp\left(-\frac{q\phi - \beta\sqrt{E}}{k_B T}\right)$
Space-charge-limited conduction	$J \propto E\, exp\left(-\frac{E_t}{k_B T}\ln\left(\frac{qH_t d}{2\,\varepsilon_0\varepsilon_r\,E}\right)\right)$

Supplementary References

1 Toma, F. M. et al. Mechanistic insights into chemical and photochemical transformations of bismuth vanadate photoanodes. *Nat. Commun.* **7**, 12012 (2016).

2 Helander, M. G., Greiner, M. T., Wang, Z. B., Tang, W. M. & Lu, Z. H. Work function of fluorine doped tin oxide. *J. Vac. Sci. Technol. A* **29**, 011019 (2011).

3 Gordon, R. G. Criteria for Choosing Transparent Conductors. *MRS Bull.* **25**, 52-57 (2000).

4 Cooper, J. K. et al. Electronic Structure of Monoclinic $BiVO_4$. *Chem. Mater.* **26**, 5365-5373 (2014).

5 Dong, W., Guo, Y., Zhang, Y., Li, H. & Liu, H. Photoelectric properties of $BiVO_4$ thin films deposited on fluorine doped tin oxide substrates by a modified chemical solution deposition process. *Int. J. of Hydrog. Energy* **39**, 5569-5574 (2014).

6 Spadafora, E. J. et al. Work function tuning for flexible transparent electrodes based on functionalized metallic single walled carbon nanotubes. *Carbon* **50**, 3459-3464 (2012).

7 Warren, S. C. et al. Identifying champion nanostructures for solar water-splitting. *Nat. Mater.* **12**, 842-849 (2013).

8 Lorenzoni, M., Giugni, A. & Torre, B. Oxidative and carbonaceous patterning of Si surface in an organic media by scanning probe lithography. *Nanoscale Res. Lett.* **8**, 75 (2013).

9 Griesser, T. et al. UV-induced modulation of the conductivity of polyaniline: towards a photo-patternable charge injection layer for structured organic light emitting diodes. *J. Mater. Chem.* **22**, 2922-2928 (2012).

10 Nevosad, A., Hofstaetter, M., Wiessner, M., Supancic, P. & Teichert, C. in *SPIE OPTO.* 8 (SPIE) (eds Ferechteh Hosseini Teherani, David C. Look, & David J. Rogers) 862618 (2013).
