# Supplementary material for: Nanoscale imaging of charge carrier transport in water splitting photoanodes
Source: Nat Commun. 2018 Jul 16;9:2597. doi: 10.1038/s41467-018-04856-8 (PMC6048052; doi:10.1038/s41467-018-04856-8)
Supplement: Supplementary file 2 — Description of Additional Supplementary Files [file 41467_2018_4856_MOESM2_ESM.pdf]

Description of Additional Supplementary Files

File Name: Supplementary Data 1
Description: Supplementary Dataset 1 includes all the data source for figures 2e-f, 3, 4.
